# Weed Host Specificity of the Aphid, *Aphis spiraecola:* Developmental and Reproductive Performance of Aphids in Relation to Plant Growth and Leaf Chemicals of the Siam Weed, *Chromolaena odorata*

**DOI:** 10.1673/031.012.2401

**Published:** 2012-02-10

**Authors:** B.K. Agarwala, Jhuma Das

**Affiliations:** Ecology and Biodiversity Laboratories, Department of Zoology, Tripura University, Suryamaninagar, Tripura West 799 022, India

**Keywords:** Apterous aphids, reaction norms, host plant growth stages

## Abstract

Density, distribution, and nutritional quality of plants are the causal basis of host plant selection in aphids. Nutritional qualities of a plant vary according to its growth stage and also in response to seasonal variation. How host plant growth stages shape aphid performance was studied in *Aphis spiraecola* Patch (Homoptera: Aphididae) on the perennial Siam weed, *Chromolaena odorata* (L.) King and Robinson (Asterales: Asteraceae). This plant species is the preferred host in the hot and humid tropical parts of northeast and southern India. Variations in developmental and reproductive performances in apterous viviparous female aphids were recorded in relation to differences in leaf chemicals in different growth stages of *C. odorata.* Aphids reproduced at higher rates in the vegetative stage of *C. odorata* when developmental time was shortest, and fecundity was higher in a longer reproductive time. Intrinsic rate of increase and net reproductive rate were also recorded to be higher in the vegetative stage of the weed host. In the vegetative stage, leaves contained higher quantity of proteins and nitrogen, which are vital for insect reproduction. Results of this study have demonstrated that A *spiraecola* showed synchronization of its developmental and reproductive performances to growth stages of *C. odorata*, which occur in high abundance in the study area.

## Introduction

Aphids realize their great reproductive potential through parthenogenesis and viviparity, and synchronize their life cycle to growth stages and phenology of host plants (Dixon 1988; Agarwala and Datta 1999). Ability of aphids to produce winged morphs in response to crowding or deterioration of food quality of their host plants help these insects to quickly distribute far and wide in search of suitable food plants. Even on the same host plant, populations can build up, and developmental and reproductive performance of aphids can vary in response to host quality changes in growth stages of their hosts (Honek 1987; Sequeira and Dixon 1996; Honek and Martinkova 1999). In general, aphids that live on herbs assimilate more energy and attain more growth per unit of phloem sap consumed than the aphids that live on trees (Llewellyn 1982). *Aphis spiraecola* Patch (Homoptera: Aphididae) occur worldwide on a large number of taxonomically unrelated plants including economically important crops like citrus, cocoa, egg plants, and anona (Kranz et al. 1977; Raychoudhuri 1983; Blackman and Eastop 1984; Pfeiffer et al. 1989).

In the hot and humid environment of south and northeast India, the perennial Siam weed, *Chromolaena odorata* (L.) King and Robinson (Asterales: Asteraceae), is the most common host plant of *A. spiraecola* (Raychoudhuri 1980). It is an aggressive perennial weed that occurs widely in agriculture, plantations, forests, and degraded lands, and is found to be the exclusive host of *A. spiraecola* in the area of study at Tripura (23° 45′ N, 91° 30′ E), a province in the southern part of northeast India adjoining Bangladesh (Das and Agarwala 2010). In a year round study, it was recorded that this wild shrub propagates by sexual as well as vegetative reproduction. Sexual reproduction starts when the plant is one year old. Flowering coincides with the onset of dry season (October-December) and lasts for 3–5 months. Seed germination is favored by water and light, and therefore takes place at the start of the rainy season (March-April). Active vegetative growth occurs in hot and humid months (May-September) and is followed by flowering. Seeds reach maturity in March. Shoot mortality is common in the dry season. Regeneration of dry shoots mainly occurs from sprouts of the underground roots and de— shooted stumps in the rainy season (May-September). Some regeneration also occurs during January-February. The weed host and the aphid population were both found to occur throughout the year in hot and humid environment (Das and Agarwala 2011).

This study aimed to investigate the response of acyclical populations of *A. spiraecola* to changes in different growth stages of its weed host plant, and the maintenance of its population year—round in this part of the world. This was investigated in the context of aphid— host interaction with respect to effects of biochemical properties of apical leaves in growth stages (feeding sites) on development and reproduction of apterous aphids of *A. spiraecola.*

## Materials and Methods

### Insect material

Live fourth instar apterous viviparous females of field collected *A. spiraecola* were used to raise stock culture on seedling (25–30 days old), vegetative (90–100 days old), flowering (260–280 days old), and seed maturation and dispersal (300–360 days old) stages of *C.* *odorata* under greenhouse conditions (22 ± 2 °C, 65% RH, 16:8 L:D) (Agarwala et al. 2009; Bhadra and Agarwala 2010). Several replicates of aphid rearing were maintained simultaneously to get an uninterrupted supply of aphids for treatments. A large number of *C. odorata* plants were grown to their respective growth stages to get an uninterrupted supply of growth stage—specific plants in treatments. Only one (1) fourth instar nymph from the stock culture (considered to represent first generation) was placed on individual plants of corresponding growth stages in order to raise a clone. Ten replicates were used for each of the four host growth stages in greenhouse conditions. Aphids from the stock culture were assigned to treatments randomly. Thus, 40 replicates representing as many aphid clones were used in four treatments. Aphids on individual plants in different growth stages were put in ventilated cages of appropriate size in greenhouse conditions. Individual plants in each growth stage were changed with fresh ones of the corresponding age group at suitable intervals to overcome aging effects. As a result, there was a steady supply of aphids of the same genetic lineage for each replicate of the four growth stages of the host plant. Only apterous aphids were used in the study, because this morph is a true colonizer of host plant, in contrast with the alate or winged aphids, which are dispersal morphs ready to take off in search of new suitable host plants. Fourth instar nymphs of the second generation from laboratory clones were placed individually on leaves detached at the petiole joint from the apical part of the potted plants of respective growth stages. These aphids were individually kept in leaf cages (Blackman 1987) in an environmental chamber at 22 ± 1 °C, 65% RH, and 16:8 L:D, and were allowed to develop into adults and reproduce. On each leaf, one newborn nymph of the third generation was retained and allowed to grow to maturity. 20 replicates were used in each growth stage simultaneously. Leaves were replaced by fresh ones every 12 hours to maintain the vigor of the culture. Previous studies on aphid—host plant interaction have shown that freshly detached leaves with their petiole inserted in sponges soaked in water in leaf cages did not affect the performance of a few aphids for 12 hours (Blackman 1987).

### Developmental and reproductive performance of *A.* *spiraecola*

The following attributes of development and reproduction were studied: birth weight (BW), adult weight at the final molt (AW), developmental time from the birth of a nymph to its final molt (DT), generation time from the birth of a nymph to the time of onset of reproduction by this nymph (GT), reproductive time from the first birth of nymph by an apterous female to the last birth of nymph (RT), fecundity (F), duration of the final molt (D_FM_), and the duration between final molting and birth of the first progeny (D_1st PG_). Net reproductive rate (*R*_0_), which is the multiplication rate of an organism per generation in terms of number of female offspring produced by a cohort of females, was calculated by the formula (Krebs 1985):


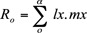


where *l*_x_ is the proportion of females surviving and *m*_x_ is the number of female offspring produced per female during its reproductive time. Intrinsic rate of increase (R_max_), which is a measure of the rate of increase of a population under controlled conditions, was calculated by the formula (Krebs 1985):


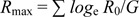


where *G* is the mean length of generation determined by the formula


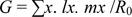


where x is the age of the adult female. Weights of aphids were taken in a Mettler electronic balance (Mettler Toledo, www.us.mt.com) sensitive to 2 µg.

### Analysis of *C. odorata* leaf chemicals

Quantitative analysis of primary metabolites present in leaf tissues was determined by dry weight, namely total nitrogen (%) (Lang 1958), total proteins (mg/gm) (Lowry et al. 1951), and total soluble carbohydrates (Yemm and Wills 1954). Ten similarly—aged apical leaves of plants in seedling, vegetative, flowering, and seed maturation stages of *C. odorata* were used from the greenhouse.

### Data analysis

Data of developmental and reproductive performance of *A. spiraecola* clones from different growth stages (treatments) of *C. odorata* were subjected to analysis of variance (ANOVA) for effect of interactions (aphid clone × plant growth stage or leaf chemicals). Data recorded in percentages were arcsin square root transformed before statistical analysis. Differences in mean values between treatments were compared by Tukey's multiple range test using Statistica computer program(www.statsoft.com). Correlation coefficient and regression analysis of relationships using best—fit curves between performance of aphid clones and leaf chemicals of plant growth stages were determined. The probability level for all statistical tests was 0.05.

**Table 1.**
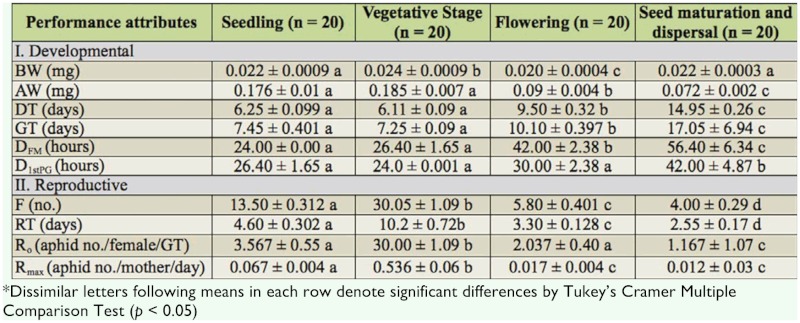
Mean ± SEM values of developmental and reproductive performance of apterous female morph *of Aphis spiraecola* clones reared on different growth stages of *Chromolaena odorata* leaves.

## Results

### Effect of plant growth stages on developmental and reproductive performance of *A.* *spiraecola*

The fresh weight of newborn nymphs (BW) from aphid clones reared on flowering stage was found to be the lowest (0.020 ± 0.0004 mg), and those from aphid clones reared on vegetative stage was found to be the highest (0.024 ± 0.0009 mg) (*F* = 112.15; *df* = 3, 76;*p* < 0.01). Mean weight of aphids at final molting (AW) in clones reared on seedling and vegetative stages was greater by 1.9 times and 2.04 times, respectively, than those reared in flowering stage, and was greater by 2.5 and 2.6 times, respectively, to the aphids reared on seed maturation and dispersal stage (*F* = 72.93; *df* = 3, 76;*p* < 0.01) (Table 1).

Development time (DT) and generation time (GT) were the longest in aphid clones reared on seed maturation and dispersal stage, and the shortest in aphids from seedling stage. The difference between the longest and shortest durations was recorded to be 58.19% in development time and 57.4% in generation time (DT: *F* = 338.58, *df* = 3, 76, *p* < 0.01; GT: *F* = 401.26, *df* = 3, 76, *p* < 0.01). No difference in developmental time and generation time was recorded in aphid clones reared on leaves of seedling and vegetative stages of *C. odorata* (Table 1).

The time taken to molt from fourth instar to adult stage (D_FM_) was longer in aphids reared on flowering (42.00 ± 2.38 hours) and seed maturation (56.40 ± 6.34 hours) stages than those reared on seedling (24.00 ± 0.0 hours) and vegetative (26.40 ± 1.65 hours) stages. Time taken by an adult apterous aphid to produce first progeny (D_1st PG_) was shorter in seedling (26.40 ± 1.65 hours) and vegetative (24.00 — 0.001 hours) stages than in flowering (30.00 ± 2.38 hours) and seed maturation (42.00 ± 4.87 hours) stages.

Mean fecundity (F) recorded in *A. spiraecola* clones from different plant growth stages was found to be significantly different (*F* = 370.35, *df* = 3, 76, *p* < 0.01). Aphids fed on leaves of vegetative stage produced, on average, 86.7% more offspring (30.05 ± 1.09 aphids) than those reared in seed maturation stage (4.00 ± 0.29 aphids). Reproductive time (RT) was the longest in aphids reared on vegetative stage (10.2 ± 0.72 days) in comparison to aphids reared on other growth stages (*F* = 73.18, *df* = 3, 76, *p* < 0.01). Aphids that fed on seed maturation stage showed the shortest reproductive time (2.55 ± 0.17 days).

**Table 2.**
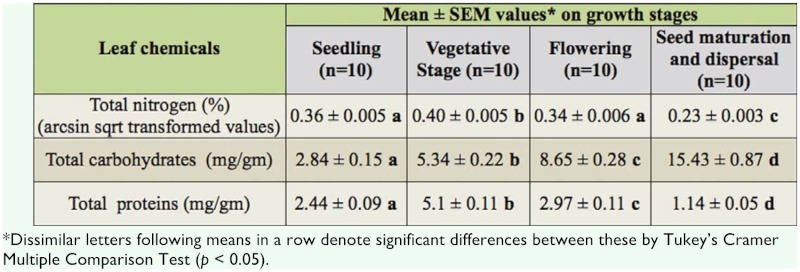
Mean ± SEM values of leaf chemicals present in different growth stages of *Chromolaena odorata.*

Net reproductive rate (*R*_0_) and intrinsic rate of increase of aphids (*R*_max_) reared in vegetative stage were highest (net reproductive rate = 25.79 ± 3.94 aphids/female; intrinsic rate of increase = 0.536 ± 0.06 aphids/mother/day) than those reared on other growth stages of the host (*R*_0_: *F* = 107.34, *df* = 3, 76, *p* < 0.01; *R*_max_: *F* = 588.39, *df* = 3, 76, *p* < 0.01). Thus, an apterous aphid that fed on vegetative stage produced, on average, 12.66, 7.23, and 21.5 times more aphids than one that fed on flowering, seedling, and seed maturation stages, respectively. Intrinsic rate of increase of aphids on vegetative stage was found to be 31.53, 8.00, and 44.7 times higher than that fed on seedling, flowering, and seed maturation stages, respectively (Table 1).

### Analysis of *C. odorata* leaf chemicals

As percent of dry weight of leaves (arcsin square root transformed values), the quantity of total nitrogen present in vegetative (0.40 ± 0.005) and seedling (0.36 ± 0.005) stages was higher than that recorded in leaves of flowering (0.34 ± 0.006) and seed maturation (0.23 — 0.003) stages (*F* = 65.24, *df* = 3, 36, *p* < 0.01) (Table 2). The highest quantity recorded in vegetative stage was 3.06 times more than the lowest quantity of soluble nitrogen present in seed maturation stages. In contrast, quantity of soluble carbohydrates was found to be higher in leaves of flowering (8.65 — 0.28 mg/gm of leaf) and seed maturation (15.43 — 0.87 mg/gm of leaf) stages than that recorded in leaves of seedling (2.84 — 0.15 mg/gm of leaf) and vegetative (5.34 — 0.22 mg/gm) stages (*F* = 235.81, *df* = 3, 36, *p* < 0.01). The highest quantity of carbohydrates recorded in the seed maturation and dispersal stage was 5.4 times more than that recorded in seedling stage. Mean quantity of proteins was highest in leaves of vegetative stage (5.10 — 0.11 mg/gm of leaf) and lowest in the seed maturation and dispersal stage (1.14 — 0.05 mg/gm of leaf).

### Relationship between developmental and reproductive performance of *A. spiraecola* and leaf chemicals in growth stages of *C. odorata*

**Figure 1.**
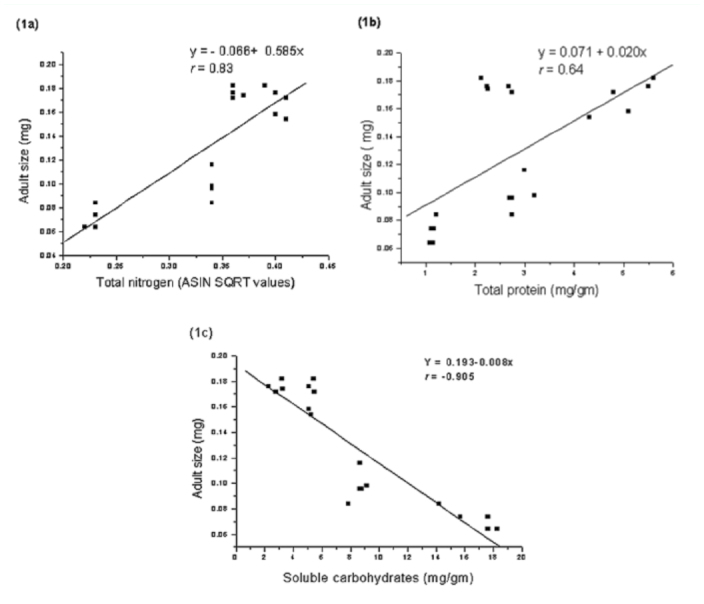
Regression relationship with fitted curves between adult size of *Aphis spiraecola* and leaf chemicals estimated at seedling, vegetative, flowering, and seed maturation stages of *Chromolaena odorata:* (a) with total nitrogen, (b) with total proteins, and (c) with soluble carbohydrates. High quality figures are available online.

**Figure 2.**
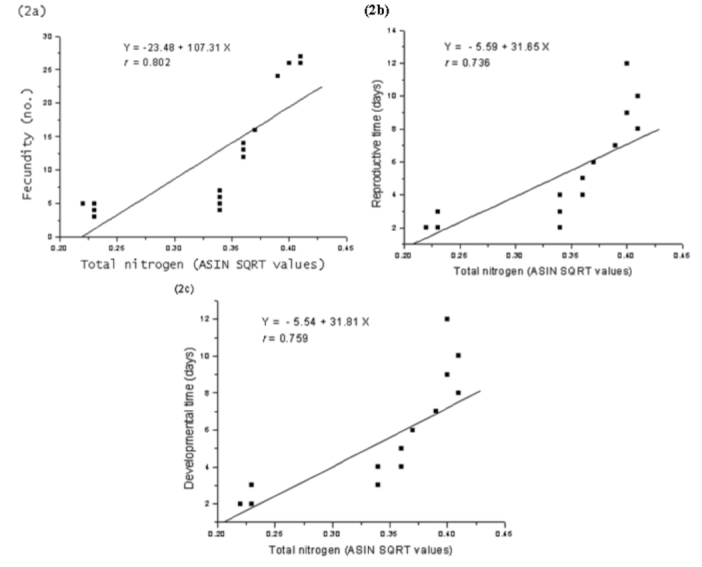
Regression relationship with fitted curves between (a) fecundity, (b) reproductive time, and (c) developmental time of *Aphis spiraecola* and total nitrogen of leaf estimated at seedling, vegetative, flowering, and seed maturation stages of *Chromolaena odorata.* High quality figures are available online.

**Figure 3.**
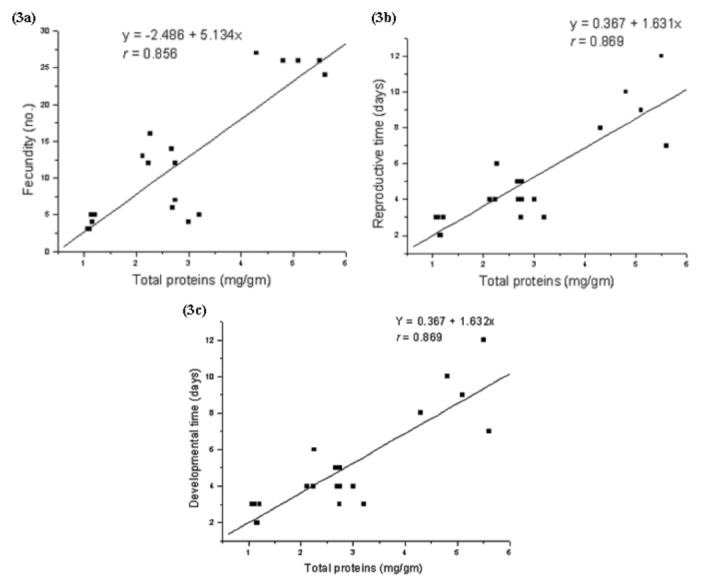
Regression relationship with fitted curves between (a) fecundity, (b) reproductive time, and (c) developmental time of *Aphis spiraecola* and total protein of leaf estimated at seedling, vegetative, flowering, and seed maturation stages of *Chromolaena odorata.* High quality figures are available online.

**Figure 4.**
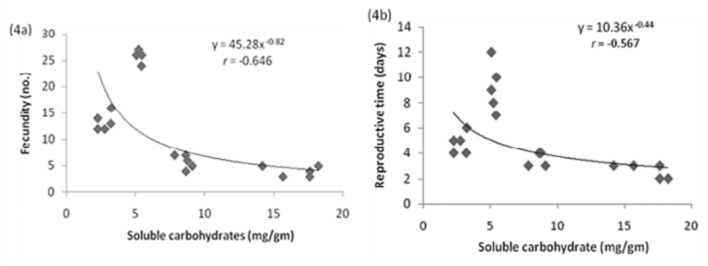
Regression relationship with fitted curves between (a) fecundity and (b) reproductive time of *Aphis spiraecola* and soluble carbohydrate of leaf estimated at seedling, vegetative, flowering, and seed maturation stages of *Chromolaena odorata.* High quality figures are available online.

**Figure 5.**
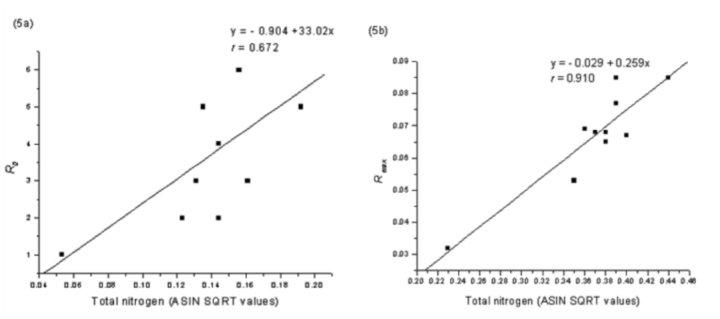
Regression relationship with fitted curves between (a) R_0_ and (b) R_max_ *of Aphis spiraecola* and total nitrogen of leaf estimated at seedling, vegetative, flowering, and seed maturation stages of *Chromolaena odorata.* High quality figures are available online.

Size of adult aphids showed strong linear relationship to total nitrogen content of leaf tissues (y = -0.066 + 0.968x; *r* = 0.83; Figure 1a) but the response was weak to total proteins (y = 0.071 + 0.020x, *r* = 0.64; Figure 1b). In contrast, aphid size showed inverse relationship to total soluble carbohydrates present in leaves (y = 0.193 - 0.008x, *r* = 0.905; Figure 1c). Fecundity, reproductive time, and developmental time of aphids showed positive relationships to total nitrogen content (slope values: fecundity = 107.31; reproductive time = 31.65; developmental time = 31.81; Figures 2a, 2b, 2c), low nitrogen concentration in the seed maturation stage (Table 2) had a negative effect on aphid performance (intercepts: fecundity = -23.48; reproductive time = -5.59; developmental time = -5.54), but higher nitrogen concentration in leaves of seedling, vegetative, and flowering growth stages (Table 2) had corresponding influence on aphid performance. Aphid fecundity, reproductive time, and developmental time also showed linear responses to quantity of leaf proteins (Figures 3a, 3b, 3c) but nonlinear responses of inverse order occurred to soluble carbohydrates content of leaves (Figures 4a, 4b); at lower carbohydrate content, fecundity and reproductive time declined rather sharply but the rate of decline decreased at higher carbohydrate content (fecundity: y = 45.28x-^0.82^; reproductive time: y = 10.36x-^0.44^). Development time of aphids showed weaker relationship to carbohydrate content of leaves (y = 8.91 + 0.07x; *r* = 0.28). Net reproductive rate and intrinsic rate of increase of *A. spiraecola* increased linearly in relation to nitrogen content of leave tissues (Figures 5a, 5b), but these showed weak relationship to proteins and carbohydrates (*R*_0_*:* proteins, *r* = 0.156; carbohydrates, *r* = 0.25) (*R*_max_: proteins, *r* = 0.43; carbohydrates, *r* = 0.23). Mean relative growth rate, however, did not show a definite relationship to any of the leaf chemicals studied (total nitrogen: *r* = 0.12, proteins: *r* = 0.016, carbohydrates: *r* = -0.317).

## Discussion

In this study, changes in quantity of leaf nitrogen in different growth stages of *C. odorata* was found to affect the performance of *A. spiraecola.* Apterous adult aphids were heavier and produced significantly more offspring in longer reproductive time. Nymphs developed faster when fed on leaves in seedling and vegetative stages, which contained a higher quantity of nitrogen. Intrinsic rate of increase and net reproductive rate were also recorded to be higher in the vegetative stage of *C. odorata.* Aphids reared on leaves in the seed maturation stage took a longer time to molt from the fourth instar stage to the adult stage, and those reared on seedling, or vegetative, stage leaves took less time to produce the first progeny feeding on leaves of seedling or vegetative than those that fed on leaves of flowering or seed maturation stages.

Biochemical changes in plants, considered as changes in food quality for aphids, have marked effects on the development, reproduction, and longevity of aphids (Dixon 1985a). With increase in plant age, the content of proteins, carbohydrates, and nitrogen can vary (van Emden 1969; van Emden and Bashford 1971; Staley et al. 2010). Young leaves typically have two to four times more nitrogen than mature leaves (Coley and Aide 1991; Karle et al. 2002) and influence the food choice for both chewing and sucking insects (Mattson and Scriber 1987). Any change in the developmental patterns of young leaves that reduce the concentration of nitrogen reduces the reproductive performance of herbivory (Moran and Hamilton 1980; Neuvonen and Haukioja 1984; Marutani and Muniappan 1991). Higher quantity of nitrogen and proteins are known to enhance reproductive performance of herbivores (Awmack and Leather 2002; Staley et al. 2010). Several studies on aphids have reported the way in which a developing plant can affect individual aphids. Leather and Dixon (1981) found significant differences in developmental time and fecundity of *Rhopalosiphum padi* reared on pot grown plants of different developmental stages.

Poor performance by aphids on mature plants, as documented in this study, has been recorded for several other aphid species (van Emden 1969; van Emden and Bashford 1971; Williams 1995). Several studies have also recorded that leaves with lower nitrogen cause lower herbivore growth and a reduction in adult size and fecundity (Raupp and Denno 1983; Readek and Cates 1984; Brewer et al. 1985). This is particularly true of insects like aphids that feed on phloem sap. The amount of nitrogen transported through the phloem is an important component for the growth, development, and reproduction of young aphids (Dixon 1985b; Lightfoot and Whiteford 1987). Plants that show low nitrogen content are not selected by herbivores in general, and homopteran insects in particular (Chen et al. 1997; Agarwal 2001).

As phloem feeders and obligatory parasites of plants, aphids seek association with hosts that occur in high abundance in order to secure survival and reproduction of individuals generation after generation (Dixon 1988; Powell et al. 2006; Bhadra and Agarwala 2010). In the patchy environment of host distribution, especially in the tropics, the time required to search for a favorable host for a short—lived aphid is important (Agarwala and Bhattacharya 1995; Fenton et al. 2009). Plant diversity and distribution vary from region to region in relation to latitudinal gradient, climate, and physical heterogeneity of an area or region (Ricklefs and Miller 2000). Host selection in aphids, therefore, depends on the spatial dynamics of host distribution and the ability of these insects to find a host in limited time using physical and chemical cues (Muller 1985; Klingauf 1988; Powell et al. 2006). Occurrence of host races or biotypes in relation to patchy distribution of host plants has been recorded in several aphid species (De Barro et al. 1995; Agarwala 2007). Polyphagous aphids like *Aphis gossypii*, *A. fabae*, *Lipaphis pseudobrassicae*, *Myzus persicae*, and *Scizaphis* are known to occur by several host—specialized races from different parts of the world (Guldemond et al. 1994; Vanlerberghe-Masutti and Chavigny 1998; Peppe and Lomonaco 2003), and each of these races differs in growth and reproductive fitness (Nikolakokis et al. 2003; Gorur et al. 2007; Agarwala and Das 2007; Agarwala et al. 2009).

In the case of *A. spiraecola*, its common hosts like citrus, spirea, maple, annona, and cocoa occur in low abundance in this part of the world. Therefore, selection of *C. odorata* as the preferred host by *A. spiraecola* in this part of Asia could be primarily attributed to occurrence of this host in high abundance in open fields, farmlands, and plantation crops throughout the year. Populations of *A. spiraecola* feeding on *C. odorata* seem to have developed a proximate relationship with host growth stages. Acceptance of a novel host greatly facilitates divergence and reproductive isolation between aphid populations preferring and accepting different host plants (Caillaud and Via 2000). A study on genotypic differences in populations of *A. spiraecola* from different host plants could be rewarding.
